# High-Intensity Intermittent Exercise and its Effects on Heart Rate Variability and Subsequent Strength Performance

**DOI:** 10.3389/fphys.2016.00081

**Published:** 2016-03-04

**Authors:** Valéria L. G. Panissa, Cesar C. Cal Abad, Ursula F. Julio, Leonardo V. Andreato, Emerson Franchini

**Affiliations:** ^1^Department of Sport, School of Physical Education and Sport, University of São PauloSão Paulo, Brazil; ^2^NAR—Nucleus of High Performance in SportSão Paulo, Brazil

**Keywords:** concurrent training, recovery interval, maximum number of repetitions, parasympathetic reactivation, muscle strength

## Abstract

**Prupose:** To investigate the effects of a 5-km high-intensity interval exercise (HIIE) on heart rate variability (HRV) and subsequent strength performance.

**Methods:** Nine trained males performed a control session composed of a half-squat strength exercise (4 × 80% of one repetition maximum—1 RM) in isolation and 30-min, 1-, 4-, 8-, and 24-h after an HIIE (1-min at the velocity peak:1-min passive recovery). All experimental sessions were performed on different days. The maximum number of repetitions (MNR) and total weight lifted (TWL) during the strength exercise were registered in all conditions; in addition, prior to each session, HRV were assessed [beat-to-beat intervals (RR) and log-transformed of root means square of successive differences in the normal-to-normal intervals (lnRMSSD)].

**Results:** Performance in the strength exercise dropped at 30-min (31%) and 1-h (19%) post-HIIE concomitantly with lower values of RR (781 ± 79 ms; 799 ± 134 ms, respectively) in the same recovery intervals compared to the control (1015 ± 197 ms). Inferential analysis did not detect any effect of condition on lnRMSSD, however, values were lower after 30-min (3.5 ± 0.4 ms) and 1-h (3.3 ± 0.5 ms) with moderate and large effect sizes (0.9 and 1.2, respectively) compared with the control condition (3.9 ± 0.4 ms).

**Conclusion:** Both RR and lnRMSSD seem to be associated with deleterious effects on strength performance, although further studies should be conducted to clarify this association.

## Introduction

Concurrent training (an association of aerobic and strength exercises) is performed by athletes of many sports in order to acquire the adaptations promoted by both activities (Leveritt and Abernethy, 1999; Chamari and Padulo, 2015). When preceded by aerobic exercise, especially high-intensity intermittent aerobic exercise, acute impairment of strength has been reported by various studies (Craig et al., 1991; Panissa et al., 2012; Inoue et al., 2015), which could contribute to the inhibition of long-term strength and hypertrophy gains (Sale et al., 1990; Craig et al., 1991).

An adequate recovery interval between aerobic and strength exercises can minimize and, depending on the duration, suppress the negative acute interference on strength (Bentley et al., 2000; Panissa et al., 2012). The recovery interval has been postulated as an important variable to consider when prescribing aerobic and strength exercises on the same day since when these activities were separated by an adequate recovery interval (~4–6-h) optimized long-term results were achieved (García-Pallarés et al., 2009; García-Pallarés and Izquierdo, 2011; Robineau et al., 2016).

Recently, heart rate variability (HRV)—a noninvasive tool to assess the autonomic nervous activity through the heart rate (Pierpont et al., 2000; Padulo et al., 2013)—has been suggested as a simple device to monitor the fatigue and recovery status in several kinds of activities that require both high-intensity intermittent exercise (HIIE) and strength performances (Chen et al., 2011; Attene et al., 2014; Ibba et al., 2014; Nakamura et al., 2015; Saboul et al., 2015; Thorpe et al., 2015).

Immediately acute post-exercise parasympathetic reactivation probably depends largely on the accumulation of stress metabolites in skeletal muscle and blood (Buchheit et al., 2007) while intermediate recovery (1−48-h post-exercise) of cardiac parasympathetic activity is most likely dependent on exercise-induced changes in plasma volume and the result of arterial-baroreflex stimulation (Buchheit et al., 2009). Thus, as there are several factors which can affect HRV recovery, knowledge of the vagal reactivation time course for several kinds of activities and types of physical training may help coaches and physical trainers to prescribe exercises guided by individual responses (Buchheit, 2014).

Indeed, HRV reduces immediately after high-intensity intermittent exercise and this can continue for up to 72-h before returning to basal levels, depending on the training status, and intensity of the exercise (Imai et al., 1994; Seiler et al., 2007). Recent studies have shown that day-to-day HRV variations in athletes exposed to high training loads are associated with variations in acute performance (Plews et al., 2012; Thorpe et al., 2015) and consequently HRV is considered a useful tool to measure the recovery status between training sessions. However, only one study has investigated HRV in strength training, reporting that reductions in HRV recovery resulted in decreased strength performance (Chen et al., 2011).

The effects of HIIE on HRV and on strength performance after different recovery interval durations would also help coaches and physical conditioning trainers to improve training session organization. However, to the best of our knowledge, no studies have been conducted which report the time course of HRV in concurrent aerobic (HIIE) and strength exercises with different recovery intervals between activities. Therefore, the purpose of the present study was to verify if the recovery of HRV is followed by performance recovery in strength exercise after an HIIE, using different recovery intervals between activities (30-min, 1-, 4-, 8-, and 24-h) and, consequently, if HRV is a practical tool to determine optimal recovery between aerobic and strength exercises. Thus, the main hypothesis of the present study was that the interference in strength performance (i.e., reduction in maximum number of repetitions or total weight lifted) would be followed by alterations in HRV response.

## Material and methods

### Subjects

Nine males [age: 27 ± 7 years; height: 180 ± 7 cm; body mass: 79 ± 11 kg; 1 RM half squat: 198 ± 34 kg; ${\dot{\text{V}}\text{O}}_{2\text{peak}}$:56.3 ± 8.3 (ml.kg^−1^.min^−1^); V_peak_19.8 ± 2.4 km/h], voluntarily participated in the present study after reading and signing an informed consent explaining all the risks and benefits of the present investigation. All procedures received local ethics committee approval. All individuals were nonsmokers, and none of them received any pharmacological treatments or had any type of neuromuscular disorder or cardiovascular, respiratory or circulatory dysfunction. All participants had a minimum of two consecutive years of training experience and practiced systematic aerobic and strength training with a volume of approximately 7-h per week. The subjects were instructed to abstain from any strenuous exercise for at least 48-h before each testing session. All participants were oriented to maintain their habitual sleep and food routines. Tests were conducted at an average room temperature of 27°C and the experimental session was performed in the time normally used for training.

### Study design

**Figure 1:**
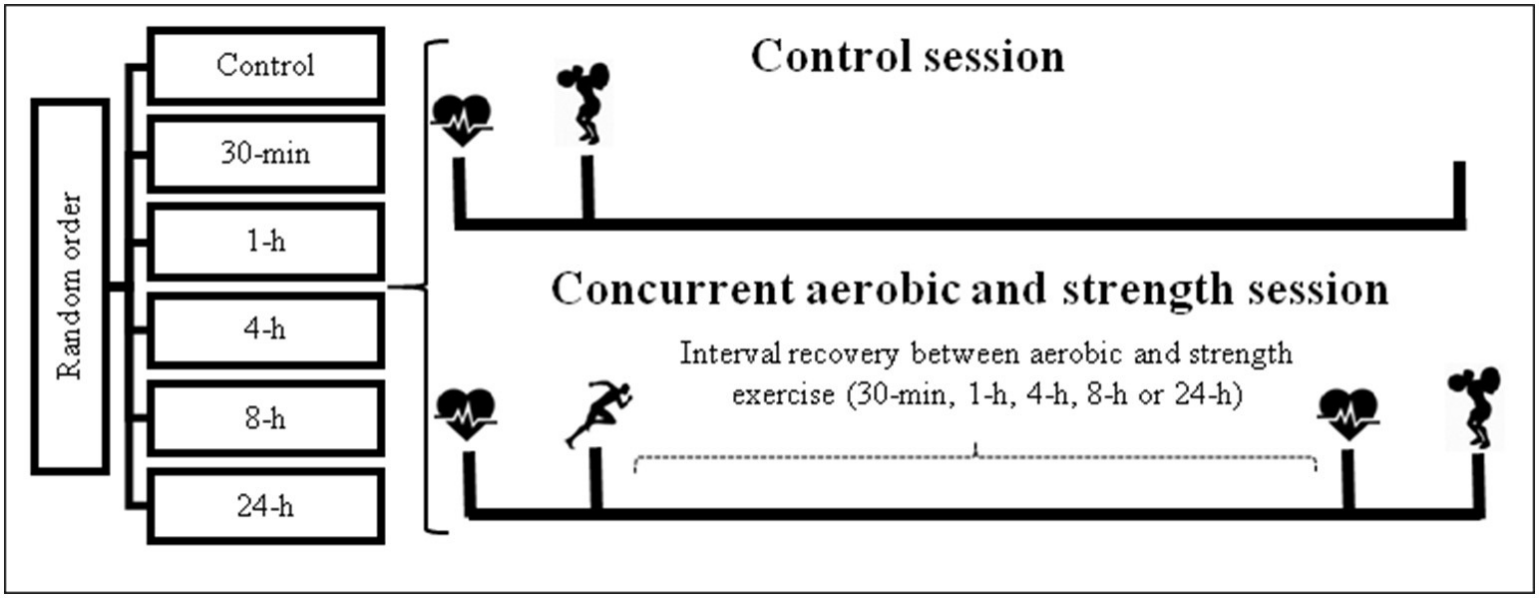
**Design of study**. 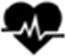, heart rate variability acquisition; 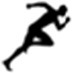, high-intensity intermittent exercise; 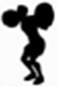 strength exercise; h, hours; min, minutes.

To investigate if the total volume performed in the strength session in athletes was impaired after an HIIE, with different recovery intervals (30-min, 1-, 4-, 8-, and 24-h), eight sessions were conducted (Figure 1). In the first session, after body mass and height measurements, athletes carried out an incremental treadmill test to volitional exhaustion to determine the peak oxygen uptake (${\dot{\text{V}}\text{O}}_{2\text{peak}}$) and peak velocity attained (V_peak_). On the same day, a maximum strength test in the half-squat exercise (one repetition-maximum, 1 RM) was conducted for familiarization. In the second session the athletes performed the 1 RM test according to previous recommendations (Brown and Weir, 2001). Next, on different days the volunteers were evaluated during six experimental sessions applied in randomized order using a random draw of numbers: one control condition strength training exercise performance (four sets of maximal number of repetitions at 80% of the 1 RM load for the half squat exercise); and five sessions composed of HIIE (1:1 min at V_peak_, totaling 5 km), followed by the strength exercise, using the same protocol applied in the control condition, but using different time intervals (30-min, 1-, 4-, 8-, and 24-h). HRV was collected prior to each session of HIIE and strength exercise. Experimental sessions were performed randomly on different days, with a minimal time interval of 3 days and a maximum of 7 days between sessions.

### Incremental test

The subjects performed an incremental treadmill test to volitional exhaustion (Panissa et al., 2015). The initial speed was set at 8 km·h^−1^. Each stage lasted 1-min and was increased by 1 km·h^−1^ per stage until the subject could no longer continue. The oxygen uptake (${\dot{\text{V}}\text{O}}_{2}$) was measured (K4b^2^ Cosmed, Rome, Italy) throughout the test and the average of the final 30-s was defined as ${\dot{\text{V}}\text{O}}_{2peak}$. The maximal velocity reached in the test was defined as the V_peak_. When the subject was not able to finish the 1-min stage, the speed was expressed according to the time spent in the final stage; V_peak_ = [velocity of penultimate stage + (time, in s, remained at the last stage/60 s)].

### Maximum strength test

Maximum dynamic strength for the half squat (1 RM) was assessed using a smith machine. The test was performed according to standard procedures (Brown and Weir, 2001). Briefly, the subjects began the test with a general warm up, consisting of cycling (70 rpm at 50 W) for 5-min, followed by two specific warm-up sets. In the first set, the athletes performed eight repetitions at 50% of the estimated 1 RM, and for the second set, they performed three repetitions at 70% of the estimated 1 RM with a 2-min interval between each set. After the specific warm-up the subjects rested for 2-min and were then allowed up to five trials to achieve the 1 RM load (i.e., maximum weight that could be lifted once with the proper technique), with a 3- to 5-min interval between trials. To better control the movement during the 1 RM test, both feet position and movement amplitude (90°) were controlled. This standardization was repeated in the subsequent experimental sessions. The intraclass coefficient correlation and standard error measurement between the familiarization session and the maximum strength test were 0.994 and 5.1 kg, respectively.

### High-intensity intermittent exercise

The athletes performed a warm-up for 5- (at 8 km·h^−1^) and 2-min later started the HIIE exercise. The HIIE consisted of a 5-km run on a treadmill performed intermittently, consisting of 1-min at V_peak_ separated by 1-min in passive recovery. This protocol was chosen as HIIE can potentiate the decrease in strength endurance performance when compared with moderate intensity exercise (De Souza et al., 2007; Painelli et al., 2014). De Souza et al. (2007) and Painelli et al. (2014) reported this effect using the same protocol as that adopted in the present study. This protocol guarantees the occurrence of interference and, consequently, assists in identifying the effects of HRV on this process.

### Strength exercise

The participants performed a general warm-up consisting of cycling (70 rpm at 50 W) for 5-min, followed by four sets of maximum repetitions at 80% of the 1 RM in the half squat in the smith machine. Each set was separated by a 2-min interval. The maximum number of repetitions (MNR) were computed and the total weight lifted (TWL) was also calculated as follows: TWL = MNR × weight lifted (De Souza et al., 2007; Panissa et al., 2012, 2015).

### Heart rate variability

At least 24-h prior to the HRV assessment, the athletes were requested not to perform any training or physical activity and maintain normal sleep and nutrition routines including coffee consumption. Although caffeine demonstrates conflicting results depending on habitual or non-habitual coffee consumption (Rauh et al., 2006; Zimmermann-Viehoff et al., 2015), the HRV assessment preceded both the HIIE and strength exercise sessions and all procedures were conducted according to previous recommendations Task Force off The European Society of Cardiology, the North American Society of Pacing Electrophysiology (1996). Prior to the warm-up the volunteer assumed the sitting position, remained quiet, eyes open, and breathing spontaneously for 10-min. In a comfortable sitting position, the beat-to-beat intervals (RR) were registered for 5-min using the cardiofrequencymeter Polar® RS800CX™(Polar Electro Oy, Kempele, Finland), which has a frequency of acquisition according to the recommendations of the Task Force off The European Society of Cardiology, the North American Society of Pacing Electrophysiology (1996 1000 Hz) and has also been used in several studies due to its validity and reproducibility (Gamelin et al., 2006; Figueiredo et al., 2015b). For HRV measurement, only the middle 2-min of the RR register were analyzed (Esco and Flatt, 2014). The time series of heart rate (tachograms) were manually filtered by visual inspection. Ectopic beats and artifacts (<5%) were automatically detected and replaced by interpolated adjacent beats by applying an adaptive filter to generate normal-to-normal (NN) interval time series. All analyses were performed using Kubios HRV software. Finally, the HRV was obtained in the time domain by the normal beat-to-beat intervals [RR (ms)] and the root means square of successive differences in the NN intervals [RMSSD (ms)], which was log-transformed (lnRMSSD) to reduce bias arising from non-uniformity of error and also to avoid outliers and simplify its analysis (Nakamura et al., 2015). The lnRMSSD was chosen as it reflects the cardiac parasympathetic modulation; is not affected by breathing frequency; is the time domain measure recommended for short-term HRV analysis; and has higher reliability than the spectral analysis index (Task Force off The European Society of Cardiology, the North American Society of Pacing Electrophysiology, 1996; Nakamura et al., 2015).

### Statistical analysis

Data normality was verified using the Shapiro-Wilk test. Rest RR and lnRMSSD (i.e., prior to HIIE and prior to strength exercise in the control condition) were analyzed separately by one-way repeated measure of mixed model followed by the Tukey's *post-hoc*. This was done in order to guarantee that the subjects initiated sessions with the same basal values. To compare the RR, the lnRMSSD before each strength session and the MNR and TWL during the strength exercise, a one-way repeated measure of mixed model was applied. When a significant difference was observed, a Dunnett's *post-hoc* test was conducted. Statistical significance was set at *p* < 0.05. The data were analyzed using SAS (version 9.3). Standardized effect sizes were also calculated by the Cohen's equations (1969) with the following threshold values: < 0.2—trivial; > 0.2 and < 0.6—small; > 0.6 and < 1.2—moderate; > 1.2 and < 2.0—large; > 2.00 and < 4.0—very large; < 4.0—nearly perfect (Hopkins, 2015). The power observed for each comparison was also presented. Using the effect size (main effect of condition), the sample size of the present study had a 98% power of detecting a difference between conditions for RR, MNR, and TWL and an 80% power to detect differences between conditions for lnRMSSD.

## Results

For MNR there was an effect of condition [*F*_(5, 40)_ = 3.19; *p* = 0.016; power observed = 0.926] with higher values in the control compared to post 30-min (*p* = 0.005) and 1-h recovery (*p* = 0.049). For TWLthere was an effect of condition [*F*_(5, 40)_ = 3.75; *p* = 0.007; power observed = 0.936] with higher values in the control compared to post 30-min (*p* = 0.001) and 1-h recovery (*p* = 0.048; all results in Table 1).

**Table 1:** **Maximum number of repetitions in four sets at 80% 1 RM in the half-squat in the control condition and after 5 km running with a different recovery interval between exercises**.

	**MNR**	***d***	**ES**	**TWL (kg)**	***d***	**ES**
Control	50 ± 14	–	–	7550 ± 1097	–	–
30 min	34 ± 11^*^	1.27	Large	5152 ± 1090^*^	2.19	Very large
1-h	40 ± 12^*^	0.77	Moderate	6140 ± 1162^*^	1.25	Large
4-h	45 ± 18	0.31	Small	6749 ± 2078	0.48	Small
8-h	42 ± 12	0.69	Moderate	6030 ± 1774	1.03	Moderate
24-h	46 ± 16	0.27	Small	6991 ± 1645	0.40	Small

*Data are mean ± standard deviation. MNR, maximum number of repetitions; TWL, Total weight lifted; d, Cohen's effect size compared with control condition; ES, qualitative ES description*.

* *different from control condition (p < 0.05)*.

Repeated measures one-way did not show any difference for any variables between pre-HIIE in all conditions. Repeated measures one-way, considering all pre-strength exercise values in each condition, demonstrated an effect for RR [*F*_(5, 32.2)_ = 6.56; *p* < 0.001; power observed = 1.00], with higher values in the control condition compared to post 30-min (*p* < 0.001), and 1-h recovery (*p* = 0.004). There was no effect for lnRMSSD [*F*_(5, 31.9)_ = 1.80; *p* = 0.141; power observed = 0.650; all results in Table 2].

**Table 2:** **Heart rate variability responses in control condition and after different recovery durations after high-intensity intermittent exercise**.

	**RR (ms)**	***d***	**ES**	**lnRMSSD (ms)**	***d***	**ES**
Control	1015 ± 197	–	–	3.9 ± 0.4	–	–
30-min	781 ± 179^*^	1.23	Large	3.5 ± 0.4	0.92	Moderate
1-h	799 ± 134^*^	1.28	Large	3.3 ± 0.5	1.32	Large
4-h	1043 ± 199	0.14	Trivial	3.7 ± 0.7	0.35	Small
8-h	964 ± 139	0.30	Small	3.8 ± 0.4	0.25	Small
24-h	1007 ± 184	0.04	Trivial	3.7 ± 0.5	0.44	Small

*Data are mean ± standard deviation. RR, beat-to-beat intervals; lnRMSSD, log-transformed of root means square of successive differences in the normal-to-normal intervals; d, Cohen's effect size compared with control condition; ES, qualitative description*.

* *, different from control condition*.

## Discussion

The present study aimed to investigate the effects of 5-km HIIE on HRV indices (RR and lnRMSSD) and subsequent strength performance, with different recovery intervals between activities (30-min, 1-, 4-, 8-, and 24-h). The main finding of the present study was that there was a reduction in strength performance until the 1-h recovery interval, which was accompanied by decreases in RR, with lower values and large effects after 30-min and 1-h compared to the control condition. In comparison to the control condition, moderate and large effects sizes were found for the lnRMSSD after 30-min and 1-h (ES = 0.92 and ES = 1.32, respectively).

Investigations into the recovery time-course of HRV after an high-intensity exercise show that, depending on certain aspects, it can take up to 72-h to return to basal levels (Chen et al., 2011). An important aspect to consider when observing HRV post-exercise is training status, since individuals with higher levels of aerobic training recover faster (Seiler et al., 2007). Therefore, when compared to studies with participants of similar training status, it was observed that the results of the present study are in accordance with previously published studies. Stanley et al. (2012), using trained cyclists, observed that RMSSD returned to pre-values 130-min after an HIIE (10-min of warm-up, followed by eight efforts of 4-min at 80% of peak aerobic power output interspersed by 1-min of active recovery at 50% of peak aerobic power output totalizing 40-min). Seiler et al. (2007) showed that in trained participants (4-h a week—handball or soccer athletes) involved in a 30-min interval exercise consisting of six 3-min efforts at a velocity eliciting 95–100% of $\dot{\text{V}}{\text{O}}_{2max}$, with 2-min of active recovery periods above the anaerobic threshold, thus HRV returned to pre-exercise values after approximately 90-min.

Although several studies have shown that some HRV indices are reduced for up to 50-min (Figueiredo et al., 2015a,b) after a strength session, only Chen et al. (2011) demonstrated reductions in strength performance derived from a decrease in HRV index. In a study by Chen et al. (2011), seven weightlifters performed weight training (2-h) and their HRV recovery and weightlifting performance (three attempts at maximal weight lifted—back squat, seated shoulder press, dead lift, and front squat) were monitored 3-, 24-, 48-, and 72-h post this strength session. Although the authors evaluated the HRV through spectral analysis in the short-term (5-min) and not in the time domain as in the present study, they reported strength decreases below baseline and after 72-h concomitantly with reductions in high frequency (HF) after 48-h, returning to baseline in 72-h; low frequency (LF) increased at 24-h and returned to baseline after 48-h.

Independently of the type of analysis (spectral or time domain), the results observed in the present study and in the study of Chen et al. (2011) indicate that starting a strength exercise with decreased HRV could be associated with a reduction in strength performance. It is necessary to highlight some differences in the experimental protocols, given that Chen et al. (2011) observed the time-course of HRV pre and post high volume strength exercises, being that these measurements occurred sequentially and were repeated four times (3-, 24-, 48-, and 72-h), whereas in the present study, the measurements were performed on different days, eliminating the cumulative effect. HRV recovery is generally performed on the same day (time course based on a single pre-value); however, in this case, we did not have access to the strength performance during the experimental trials on the same day, due to accumulation of fatigue in each strength test.

Concerning the association between HRV and concurrent exercises (deleterious effect on strength when preceded by aerobic exercise), this is the first study to investigate the HRV after HIIE with subsequent analysis of strength performance. The mechanisms responsible for the acute interference effect are not fully elucidated, although it is known that residual fatigue after aerobic exercise can involve metabolic and inflammatory alterations (Inoue et al., 2015), as well as alterations in recruitment of motor units (Bentley et al., 2000).

The maintenance of training volume (TWL) is an important variable related to strength gains (Robbins et al., 2012; Sooneste et al., 2013). Moreover, acute impairment in strength due to previous aerobic activity has been highlighted, resulting in long-term impairments in strength, and hypertrophy gains (Sale et al., 1990; Craig et al., 1991). To guarantee the quality of the mechanical stimulus and to avoid the acute interference on strength performance a long recovery interval between activities is recommended to maintain the training volume (García-Pallarés et al., 2009; García-Pallarés and Izquierdo, 2011).

Thus, the strategy of using HRV to evaluate the recovery status in order to suppress acute interference could be useful for coaches; however, other studies are needed to confirm this association, as well as the relationship with other biochemical or psychophysiological recovery markers.

One possible limitation of the present study is the small sample size. However, analysis of the effect sizes tends to minimize bias of small samples, allowing researchers to present the magnitude of effects in a standardized metric and communicate the practical significance of their results (Lakens, 2013). Additionally, this study used a repeated measurement design with random determination of the intervals between HIIE and the strength exercise.

In conclusion, the present study showed that aerobic and strength trained men who performed a high-intensity intermittent running exercise presented compromised strength endurance performance up to 1-h after the HIIE, with concomitant decreases in RR and lnRMSSD. After a 4-h recovery interval the interference of HIIE on strength endurance performance was suppressed and HRV returned to basal values. Hence, coaches and trainers should consider using HRV to monitor the recovery status of participants in order to avoid acute interference, preserving the mechanical stimulus and, consequently, long-term strength gains. Therefore, considering that after an acute bout of HIIE the decrement in strength occurred up to 1-h, we suggest performing strength training with interval recovery superior than this time.

Moreover, further studies should be conducted using recovery intervals in a time window between 1- and 4-h to observe the first time that HRV is recovered and whether this occurs concomitantly with the suppression of interference in strength. The relation between fatigue and recovery HRV should be explored to help in the elucidation of the mechanisms behind acute interference in strength. Finally, long-term studies could confirm whether minimizing acute interference using recovery interval and monitoring the HRV in acute sessions result in the optimization of long-term strength and hypertrophy gains.

## Author contributions

Conceived and designed the experiments: VP, UJ, EF; Performed the experiments: VP, UJ, LA; Analyzed the data: VP, CCA, LA, EF; Wrote the paper: VP, CCA, UJ, LA, EF.

### Conflict of interest statement

The authors declare that the research was conducted in the absence of any commercial or financial relationships that could be construed as a potential conflict of interest.
